# Consistency With and Disengagement From Self-monitoring of Weight, Dietary Intake, and Physical Activity in a Technology-Based Weight Loss Program: Exploratory Study

**DOI:** 10.2196/33603

**Published:** 2022-02-18

**Authors:** Chelsea A Carpenter, Abraham Eastman, Kathryn M Ross

**Affiliations:** 1 Department of Clinical and Health Psychology College of Public Health and Health Professions University of Florida Gainesville, FL United States

**Keywords:** self-monitoring, adherence, weight loss, digital tools, mobile phone

## Abstract

**Background:**

Digital self-monitoring tools offer promise to improve adherence to self-monitoring of weight and weight-related behaviors; however, less is known regarding the patterns of participant consistency and disengagement with these tools.

**Objective:**

This study characterizes the consistency of use and time to disengagement with digital self-monitoring tools during a 6-month weight loss intervention and investigates whether the provision of phone-based intervention improved self-monitoring adherence.

**Methods:**

Participants were 54 adults with overweight or obesity (mean age 49.6 years, SD 12.4 years; mean BMI 32.6 kg/m^2^, SD 3.2 kg/m^2^) enrolled in a pilot trial assessing the impact of self-monitoring technology (Fitbit Zip, Aria scale, and smartphone app), with and without additional interventionist contact, on weight loss. All participants received weight loss education and were asked to self-monitor weight, dietary intake, and physical activity daily throughout the 6-month program. Consistency was defined as the number of weeks that participants adhered to self-monitoring recommendations (7 out of 7 days). Disengagement was defined as the first of 2 consecutive weeks that the 7-day self-monitoring adherence goal was not met. Wilcoxon signed-rank tests were used to examine differences in consistency and disengagement by behavioral targets. *t* tests (2-tailed) and Cox proportional hazards models were used to examine whether providing additional interventionist contact would lead to significant improvements in consistency and time to disengagement from self-monitoring tools, respectively. Linear regressions were used to examine associations between consistency, time to disengagement, and weight loss.

**Results:**

Participants consistently self-monitored physical activity for more weeks (mean 17.4 weeks, SD 8.5 weeks) than weight (mean 11.1 weeks, SD 8.5 weeks) or dietary intake (mean 10.8 weeks, SD 8.7 weeks; *P*<.05). Similarly, participants had a significantly longer time to disengagement from self-monitoring of physical activity (median 19.5 weeks) than weight (4 weeks) or dietary intake (10 weeks; *P*<.001). Participants randomized to receive additional interventionist contact had significantly greater consistency and longer time to disengagement for self-monitoring of dietary intake compared with participants who did not (*P*=.006); however, there were no statistically significant differences between groups for self-monitoring of weight or physical activity (*P*=.24 and *P*=.25, respectively). Greater consistency and longer time to disengagement were associated with greater weight loss for self-monitoring of weight and dietary intake (*P*<.001 and *P=*.004, respectively) but not for physical activity (*P*=.57).

**Conclusions:**

Results demonstrated that self-monitoring adherence differed by behavioral target, with greater consistency and longer time to disengagement associated with lower-burden tools (ie, self-monitoring of physical activity). Consistent with supportive accountability theory, additional interventionist contact improved consistency and lengthened time to disengagement from self-monitoring of dietary intake. Given the observed associations between consistency, disengagement, and weight loss outcomes, it is important to identify additional methods of increasing consistency and engagement with digital self-monitoring tools.

## Introduction

### Background

Self-monitoring of weight and weight-related behaviors (eg, dietary intake and physical activity) is considered a cornerstone of evidence-based weight loss programs [1,2]. Research has consistently demonstrated that greater adherence to self-monitoring is associated with better weight loss outcomes [3,4]; however, rates of adherence to self-monitoring are often suboptimal and tend to decrease over time [5-8].

Digital self-monitoring tools, including smartphone apps, wearables, and smart scales, offer promise to improve adherence to self-monitoring. These tools reduce the time needed to complete self-monitoring records compared with traditional paper-and-pencil tools, capitalize on tools that many individuals already carry or wear throughout the day, and may be more acceptable to use in social situations [9]. Promisingly, evidence to date has demonstrated that these technologies can produce greater adherence to self-monitoring for participants in behavioral weight management programs when compared with traditional self-monitoring tools [7,10,11].

Digital self-monitoring tools also allow researchers to examine patterns in self-monitoring behavior on levels not previously possible. Before the introduction of these tools, self-monitoring adherence was typically assessed either via a self-report questionnaire (eg, with items asking participants if they self-monitored daily, weekly, monthly, or at other frequencies) or by counting days of self-monitoring reported on logs returned to study staff. Data from self-report questionnaires have known biases related to retrospective recall [12] and tend to have only moderate to poor association with measured self-monitoring, such that individuals tend to overreport self-monitoring behavior on these questionnaires [13]. Counts of self-monitoring logs may also lead to inaccurate estimates of self-monitoring; one study that used an unobtrusive monitoring device to assess when paper self-monitoring logs were opened found that participants self-reported monitoring their dietary intake more often than indicated by the monitoring device [14]. Moreover, missing data from these counts could indicate either a day that self-monitoring did not occur or a day that the self-monitoring was not reported (eg, when records or self-monitoring summary sheets were not returned to the study team). In contrast, digital tools automatically send data directly to cloud-based storage, reducing missingness from failure to self-report self-monitoring. Although this difference may seem trivial, a recent study demonstrated only moderate agreement between frequency of self-weighing as assessed by self-report logs and that by a digital smart scale that sent weights directly back to research servers [15].

Beyond the simple use of frequency of self-monitoring (ie, the number of times a person self-monitors during a specific period) as a marker of adherence, recent research has suggested that certain patterns of self-monitoring may be important. A total of 2 studies have demonstrated that, after controlling for frequency of self-monitoring, the consistency of self-monitoring (ie, the amount of time that a person self-monitors at a certain frequency) may matter [16,17]. Peterson et al [16] found that, after the end of an initial weight loss program, a higher frequency of self-monitoring dietary intake during a 12-month extended care program was associated with less weight regain only when coupled with higher consistency of self-monitoring (with consistency defined as the number of weeks wherein the participant self-monitored dietary intake at least 3 out of 7 days in a week). Brockmann et al [17] found no association between the total frequency of self-monitoring weight and weight regain during a 9-month observation period following a 3-month weight loss program but found that greater consistency of self-monitoring weight (defined as the number of weeks that participants self-weighed at least 6 or 7 days in a week) was associated with less weight regain. Importantly, both studies were conducted during *maintenance* periods following the end of initial weight loss programs; less is known regarding the associations between consistency of self-monitoring and weight loss during the initial weight loss period.

Furthermore, few studies have examined the concept of *disengagement* (eg, the point at which a person stops using self-monitoring tools as recommended), which may be particularly relevant when assessing patterns of self-monitoring with digital tools. Disengagement appears to occur after the initial novelty (eg, excitement related to the use of new technology) of digital self-monitoring tools wears off, especially when these tools are implemented without the support of a structured weight management program [18-21]. Studies of physical activity wearables have shown that most participants disengage with these devices after as little as 2 weeks [19,21] to 3 months [18]. Similarly, studies of commercial mobile health apps that allow for self-monitoring of dietary intake found that participants typically disengage with these apps after approximately a month [22,23]. Promisingly, research has demonstrated that provision of additional intervention support can improve the frequency of self-monitoring in technology-based interventions [24-26]; however, no studies have investigated the impact of this support on consistency with or disengagement from self-monitoring.

### Objectives

To address these gaps, this study aims to characterize patterns of consistency with and disengagement from self-monitoring technology for weight, dietary intake, and physical activity during a 6-month weight loss intervention. Data for this study were gathered from a randomized pilot trial (NCT01999244) that assessed the impact of digital self-monitoring tools, provided with and without additional phone-based interventionist contact, on weight loss in adults with overweight and obesity [26]. The primary aim of this study is to characterize the patterns of consistency and disengagement in the use of digital self-monitoring tools and to test the hypothesis that participants randomized to receive additional phone-based interventionist contact would demonstrate greater consistency and a longer time until disengagement compared with participants who did not receive this additional contact. As a secondary aim, we examine whether greater consistency and longer time to disengagement were associated with greater weight loss during the 6-month intervention period. Finally, as an exploratory aim, we explore whether participants re-engaged with digital tools after the first period of disengagement and descriptively explored whether patterns of re-engagement were different between groups.

## Methods

### Parent Study Design

This study was a secondary analysis of data collected from a randomized pilot study investigating the impact of digital self-monitoring tools, provided with and without additional phone-based interventionist contact, on weight loss over 6 months in adults with overweight and obesity [26]. Participants in the parent study were randomized to one of three treatment conditions: (1) standard, in which participants were asked to self-monitor dietary intake, physical activity, and weight via traditional self-monitoring tools (with the study providing a printed calorie reference book, a standard pedometer, paper records, and a standard bathroom scale if one was not already owned by the participant); (2) technology-based self-monitoring tools (TECH), in which participants were asked to self-monitor via digital self-monitoring tools (with the study providing a Fitbit Zip activity monitor, a Fitbit Aria smart scale, and access to the Fitbit website and smartphone app, which allowed participants to self-monitor dietary intake and observe the synced data from their activity monitor and smart scale); or (3) TECH plus additional phone-based intervention (TECH+PHONE), in which participants were asked to self-monitor using the same tools as participants randomized to the TECH condition but were also provided with 14 phone-based intervention sessions over the 6-month intervention period.

### Participants

Participants in the parent study were adults (aged 18-70 years) with overweight or obesity (BMIs between 27 and 40 kg/m^2^) who had access to a computer and Wi-Fi internet at home [26]. Potential participants were recruited through local advertisements and flyers and asked to complete a web-based prescreen assessment before attending an in-person orientation visit, during which detailed information about the study was provided and written informed consent was obtained. Potential participants were excluded from the parent study if they reported any physical limitations that prevented walking 402.3 meters without stopping, they were participating in another weight loss program, they were taking weight loss medication, they were pregnant or planned to become pregnant during the study period, or they had any medical conditions that would contraindicate participation in a weight loss program (eg, uncontrolled type 2 diabetes, uncontrolled hypertension, or history of coronary heart disease).

Given that only participants randomized to the TECH and TECH+PHONE conditions received digital self-monitoring technology, participants randomized to the standard group were excluded from this study. Full demographic details for the 54 participants randomized to TECH (27/54, 50%) or TECH+PHONE (27/54, 50%) have been published previously [26]. In brief, participants had an average age of 49.6 (SD 12.4) years and an average BMI of 32.6 (SD 3.2) kg/m^2^ at baseline. In addition, 87% (47/54) of the participants were identified as women, and in terms of race and ethnicity, 85% (46/54) identified as non-Hispanic White, 7% (4/54) as Hispanic, 2% (1/54) as non-Hispanic Black, and 6% (3/54) identified as another category or indicated multiple race and ethnicity categories.

### Intervention Components

All participants included in this study (ie, those randomized to the TECH and TECH+PHONE conditions) received access to the Fitbit website and smartphone app, a Fitbit Aria smart scale (which synced participants’ weight directly to the Fitbit website and smartphone app), and a Fitbit Zip (a small digital pedometer attached to participants’ waistbands and synced data directly to the Fitbit website and smartphone app). The Fitbit website and smartphone apps were used to self-monitor dietary intake and allowed participants to track the foods and beverages they consumed by searching for them in a comprehensive web-based database. This database listed dietary intake information for all items and allowed individuals to easily adjust portion sizes for logged items. Unless participants chose to opt out of emails from Fitbit, participants also received an automated summary email from Fitbit each Sunday that provided information regarding average steps per day, average number of calories consumed each day, weight change over the previous week, and updates and marketing content from Fitbit (this was an automated email sent by Fitbit and sent regardless of adherence to self-monitoring; it was not intended to serve as an intervention component but is discussed as it may have impacted adherence). No additional reminders to self-monitor were sent.

All participants received the same weight loss education content during the initial Weight Loss 101 session. During this session, participants were provided with a daily calorie goal (ranging from 1200 to 1500 kcal per day, based on their baseline weight) and were encouraged to consume <30% of calories from fat each day. Participants were also given two physical activity goals: (1) to gradually increase daily steps to achieve 10,000 steps per day and (2) to gradually increase engagement in moderate-intensity exercise (eg, walking at a brisk pace) to 250 min/week. Participants were then taught how to use the study-provided digital self-monitoring tools and practiced wearing the Fitbit pedometer and using the Fitbit smartphone app and website to self-monitor an example meal. During this time, support was provided for any participant who had difficulty using the tools. Starting the day after the Weight Loss 101 visit, participants were asked to self-monitor their weight, dietary intake, and physical activity each day. Participants who experienced technical challenges after the end of this Weight Loss 101 session were encouraged to call a study number to speak with a noninterventionist staff member or to contact Fitbit customer service directly if a higher level of support was needed.

Participants randomized to the TECH+PHONE condition also received 14 structured phone calls (8 weekly, 4 biweekly, and 2 monthly calls) with a trained behavioral weight loss interventionist. Each call started with a check-in assessing the frequency of self-monitoring weight, dietary intake, and physical activity, and progress toward goals since the previous call. Reinforcement was provided for goals that were successfully met, and structured problem-solving strategies [27] were used to address any participant-reported barriers to goal attainment. A brief discussion would then focus on a specific behavioral weight management topic (eg, goal-setting [28], stimulus control [29], seeking social support [30], and relapse prevention [31]). Each call ended with structured goal-setting for the following week, including specific goals for dietary intake, physical activity, and self-monitoring of weight, dietary intake, and physical activity. Overall, calls were anticipated to last for 10-15 minutes (with 3-5 minutes devoted to the check-in, 4-5 minutes devoted to the session topic, and 3-5 minutes focused on goal-setting). Participants randomized to the TECH condition received no additional intervention contact during the 6-month study period. Additional details regarding the Weight Loss 101 sessions and TECH+PHONE call content have been published previously [26].

### Intervention Outcomes

Intervention outcomes have been published previously [26]. Of the 80 participants randomized in the parent study, 27 (34%) were randomized to TECH and 27 (34%) to TECH+PHONE. Retention at the 6-month visit was 93% (25/27) for TECH and 89% (24/27) for TECH+PHONE, with no differences between groups. From baseline to the 6-month assessment visit, participants in the TECH group lost an average (mean) of −4.04 (SD 1.37) kg (a loss of 4.35%, SD 1.29% of baseline weight), whereas those in the TECH+PHONE group lost an average of −6.40 (SD 1.17) kg (7.37%, SD 1.29% of baseline weight), with no significant differences between groups.

### Measures

Sociodemographic characteristics were assessed at baseline via REDCap (Research Electronic Data Capture; Vanderbilt University) surveys [32]. Height was assessed at baseline by a trained research assistant, to the nearest 0.1 cm, using a wall-mounted stadiometer. Weight was assessed at baseline and 6 months by trained research assistants, to the nearest 0.1 kg, using a calibrated digital scale. For measurements of height and weight, participants were asked to remove shoes and wear only light indoor clothing, with pockets emptied.

All self-monitoring data were collected via the study-provided digital self-monitoring tools, with data pulled from the Fitbit servers via Fitabase [33], a third-party research data management platform. Even though participants could manually input physical activity and weight data, only data passively collected by the Fitbit Zip and Aria scale were used in this study’s analyses.

### Statistical Analyses

Given that all participants were instructed to self-monitor weight, dietary intake, and physical activity daily throughout the 6-month program, consistency of self-monitoring was defined as the number of weeks that participants met this goal (self-monitoring 7 out of 7 days), calculated separately for each type of self-monitoring. A valid day of self-monitoring weight was defined as a day on which at least one weight value was captured via the Fitbit Aria scale (the scale automatically filtered out weights that were likely to be from other users or pets). A valid day of self-monitoring dietary intake was defined as a day that any foods or beverages were logged via the Fitbit website or smartphone app, which is consistent with the approach used in previous studies [34,35]. Similarly, even though participants were encouraged to meet both an exercise minute and step goal, a valid day of self-monitoring physical activity was defined as a day that any steps were recorded via the Fitbit Zip (as this Fitbit version only tracked steps and did not allow for tracking of exercise or minutes of moderate- and vigorous-intensity physical activity). Disengagement was defined as the point at which a participant failed to self-monitor consistently (7 out of 7 days) for a period of 2 consecutive weeks (with the first week of the 2 weeks counted as the week in which disengagement began, calculated separately for each method of self-monitoring). A 2-week period was used as the threshold for disengagement, as it provided some allowance for brief disturbances (eg, illness or travel) that may have interrupted participant engagement without resulting in prolonged disengagement. For the exploratory aim, re-engagement was defined as any point at which a participant met the 7 out of 7-day self-monitoring goal after the initial 2-week period of disengagement.

Descriptive statistics were used to characterize consistency and time to disengagement (eg, the number of weeks before a participant disengaged with each self-monitoring tool) overall and by treatment condition. For the primary aim, Wilcoxon signed-rank tests were used to examine whether there were significant differences in consistency and time to disengagement among the different modalities of self-monitoring. In addition, independent 2-tailed *t* tests (*df*=53) were used to assess differences in consistency by treatment group, and Cox proportional hazards models were used to examine whether the time to disengagement from each self-monitoring method differed significantly between the 2 treatment groups. For the secondary aim, linear regressions were used to assess associations between consistency and disengagement and weight loss during the 6-month intervention. For the exploratory aim, descriptive statistics were used to characterize patterns of re-engagement after initial disengagement (eg, proportion of participants who re-engaged after disengaging, number of weeks until re-engagement, and number of weeks of re-engagement), and chi-square analyses and independent 2-tailed *t* tests were used to examine if there were differences in re-engagement among groups.

Analyses were conducted using an intent-to-treat approach, with multiple imputation used to manage missing 6-month weight data [26]. Because of the small sample size and absence of significant differences in demographic characteristics among groups [26], no additional variables were controlled for in the analyses. SAS (version 9.4; SAS Institute Inc) for Windows was used for descriptive statistics, linear regressions, and independent 2-tailed *t* tests. SPSS (version 26; IBM Corp) was used for the Cox proportional hazards models.

### Ethics Approval

The study was approved by The Miriam Hospital IRB (206813), and all participants gave their written informed consent before participation in the study.

## Results

### Consistency

**Figure 1:**
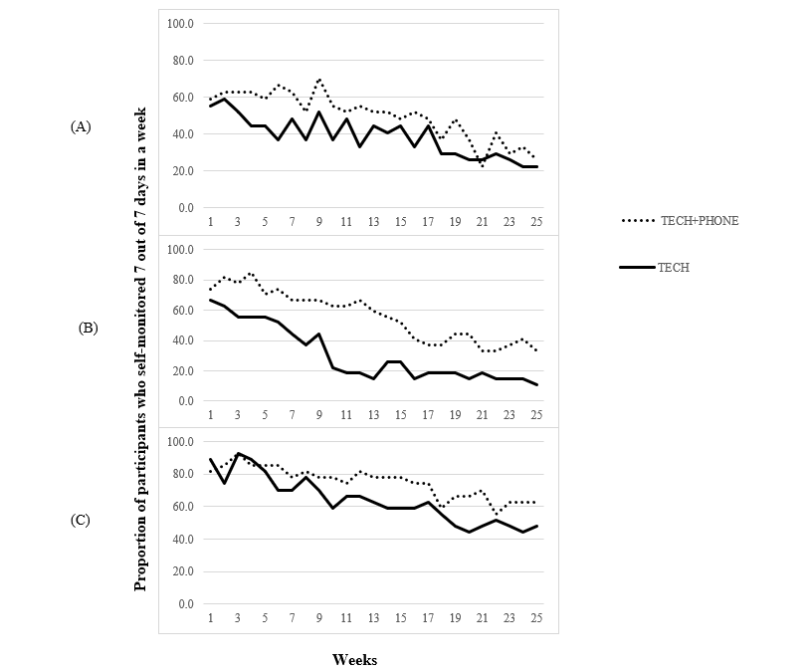
Consistency of (A) weight, (B) dietary intake, and (C) physical activity self-monitoring over time by intervention group. TECH: technology-based self-monitoring tools; TECH+PHONE: technology-based self-monitoring tools plus additional phone-based intervention.

Across the total sample of participants (N=54) randomized to either the TECH (27/54, 50%) or TECH+PHONE group (27/54, 50%), participants consistently self-monitored physical activity on more weeks (mean 17.4 weeks, SD 8.5 weeks out of 25 possible weeks) than weight (mean 11.1 weeks, SD 8.5 weeks; *P*=.02) or dietary intake (mean 10.8 weeks, SD 8.7 weeks; *P*<.001); however, there was no significant difference in consistency of self-monitoring for dietary intake versus weight (*P*=.76). Figure 1 shows the trends in the proportion of participants who consistently self-monitored weight, dietary intake, and physical activity over time, by intervention group. Approximately 57% (31/54) of the participants consistently self-monitored weight in the first week of the program, a rate that fell below 24% (13/54) by the end of the 6-month program. Similarly, approximately 70% (38/54) of the participants consistently self-monitored dietary intake during the first week of the program, whereas 22% (12/54) did so by the end of the program. Finally, 85% (46/54) of the participants consistently self-monitored physical activity during the first week of the program, and over half still did so by the end of the program.

Investigating differences between treatment groups, TECH+PHONE participants consistently self-monitored dietary intake on a greater number of weeks than TECH participants (mean 14.0 weeks, SD 8.7 weeks vs mean 7.6 weeks, SD 7.5 weeks; t_52_=−2.88; *P*=.006; Cohen *d*=0.785). There were no significant differences between TECH+PHONE and TECH in the mean number of weeks that participants consistently self-monitored weight (mean 12.5 weeks, SD 8.1 weeks vs mean 9.7 weeks, SD 10.0 weeks; *P*=.24; Cohen *d*=0.327) or physical activity (mean 18.7 weeks, SD 8.5 weeks vs mean 16.0 weeks, SD 8.4 weeks; *P*=.25; Cohen *d*=0.320).

### Disengagement

**Figure 2:**
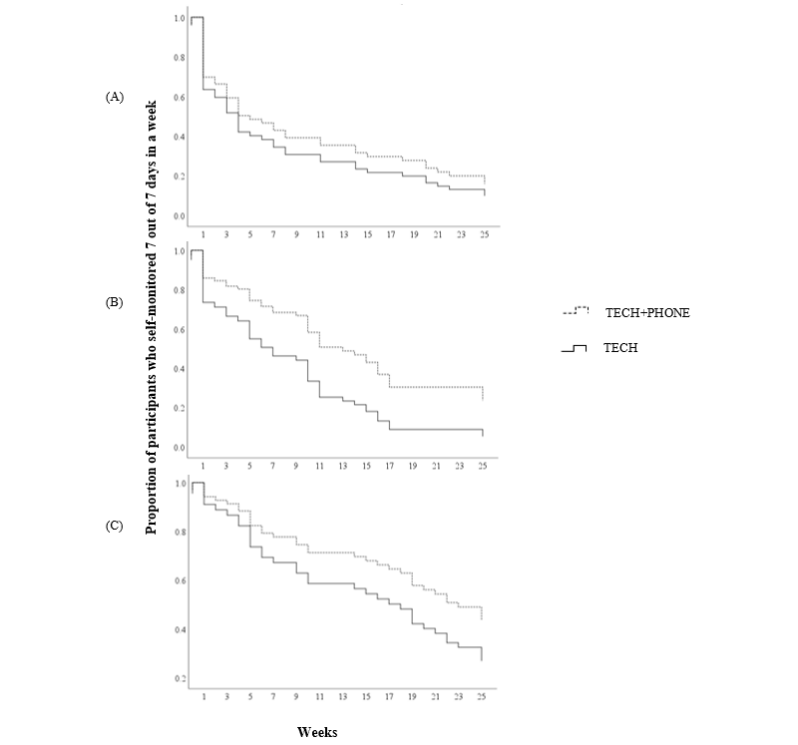
Time to disengagement from self-monitoring for (A) weight, (B) dietary intake, and (C) physical activity by intervention group. TECH: technology-based self-monitoring tools; TECH+PHONE: technology-based self-monitoring tools plus additional phone-based intervention.

Similar to consistency, participants had a significantly longer time to disengagement from self-monitoring of physical activity (median 19.5 weeks, IQR 6-26 weeks) than weight (median 4 weeks, IQR 1-18 weeks; *P*<.001) or dietary intake (median 10 weeks, IQR 3-17 weeks; *P*<.001); however, there was no significant difference in time to disengagement between dietary intake and weight (*P*=.06). Figure 2 shows survival curves for time to disengagement for self-monitoring of dietary intake, weight, and physical activity by intervention group. TECH+PHONE participants had a longer time to disengagement from self-monitoring of dietary intake compared with TECH participants (at a median of 15.5 vs 7.5 weeks, respectively; *χ*^2^_1_=5.5; *P*=.02); however, there were no significant differences in time to disengagement between TECH+PHONE and TECH participants for self-monitoring of weight (4.3 vs 11.0 weeks, respectively; *P*=.43) or physical activity (19.0 vs 25.0 weeks, respectively; *P*=.18).

### Associations Between Consistency and Disengagement and 6-Month Weight Change

There was a significant association between 6-month weight change and greater consistency of self-monitoring weight (*R*^2^=0.19; *F*_1,52_=12.34; *P*<.001), such that each additional week of consistent self-monitoring of weight was associated with an average (mean −0.33 kg, SE 0.10 kg) greater weight loss. There was also a significant association between weight change and greater consistency of self-monitoring dietary intake (*R*^2^=0.15; *F*_1,52_=8.92; *P*=.004), such that each additional week of consistent self-monitoring of dietary intake was associated with an average −0.29 (SD 0.10) kg greater weight loss. However, there was no significant association between consistency of physical activity self-monitoring and weight (*P*=.57).

Similarly, there was a significant association between time to disengagement of self-monitoring and weight loss for weight (*R*^2^=0.18; *F*_1,52_=11.12; *P*=.002) and dietary intake (*R*^2^=0.16; *F*_1,52_=9.63; *P*=.003), such that each additional week that participants remained engaged with self-monitoring of weight and dietary intake resulted in an average (mean −0.29 kg, SE 0.09 kg and mean −0.29 kg, SE 0.09 kg greater weight loss, respectively). There was no significant association between weight change during the intervention and time to disengagement for self-monitoring of physical activity (*P*=.58).

### Re-engagement

Of the 46 participants who disengaged from self-monitoring of weight, 30 (65%) re-engaged for at least 1 week. Of the 46 participants who disengaged from self-monitoring of dietary intake, 18 (39%) re-engaged for at least 1 week. Of the 35 participants who disengaged from self-monitoring of physical activity, 21 (60%) re-engaged for at least 1 week. Differences in re-engagement between the TECH and TECH+PHONE participants are presented in Table 1; the only significant difference among the groups was for the number of weeks until re-engagement for self-monitoring of physical activity, which was significantly shorter for individuals in the TECH+PHONE compared with individuals in the TECH group (*P*=.009).

**Table 1:** Re-engagement in self-monitoring for weight, dietary intake, and physical activity by intervention group (N=54)^a^.

Self-monitoring target		TECH^b^ (n=27)	TECH+PHONE^c^ (n=27)	*P* value
**Weight**				
	Participants who disengaged, n (%)	23 (85)	23 (85)	.99
	Participants who re-engaged, n (% of disengaged)	12 (44)	18 (67)	.10
	Weeks until re-engagement, mean (SD)	2.8 (0.9)	4 (3.6)	.18
	Weeks of re-engagement, mean (SD)	8 (6.2)	5.2 (5.0)	.18
**Dietary intake**				
	Participants who disengaged, n (%)	25 (93)	21 (78)	.25
	Participants who re-engaged, n (% of disengaged)	8 (30)	10 (37)	.77
	Weeks until re-engagement, mean (SD)	3.1 (1.1)	3.3 (1.8)	.82
	Weeks of re-engagement, mean (SD)	3.5 (5.0)	4.2 (3.5)	.71
**Physical activity**				
	Participants who disengaged, n (%)	20 (74)	15 (56)	.15
	Participants who re-engaged, n (% of disengaged)	13 (48)	8 (30)	.16
	Weeks until re-engagement, mean (SD)	4.6 (2.4)	2.4 (1.1)	.009
	Weeks of re-engagement, mean (SD)	4.6 (4.3)	6.5 (7.1)	.41

^a^Most periods of re-engagement were not consecutive.

^b^TECH: technology-based self-monitoring tools.

^c^TECH+PHONE: technology-based self-monitoring tools plus additional phone-based intervention.

## Discussion

### Principal Findings

This study characterized the patterns of adherence to digital tools for self-monitoring weight and weight-related behaviors within 2 technology-based weight loss programs. As expected, consistency of self-monitoring (defined as the number of weeks that participants met intervention self-monitoring goals) decreased over time. Interestingly, overall levels of consistency and rates of decline appeared to differ by target outcome, such that adherence to self-monitoring of weight and dietary intake declined more substantially than physical activity over the 6-month period. Consistency and time to disengagement (defined as a 2-week period of nonadherence to self-monitoring goals) were also significantly different between treatment groups for dietary intake, such that the provision of additional phone-based interventionist contact led to significantly greater consistency and a longer time to disengagement from self-monitoring of dietary intake; however, the provision of this additional support did not significantly impact consistency or time to disengagement for self-monitoring of weight or physical activity. The results also demonstrated that, for both weight and dietary intake, greater consistency of self-monitoring and longer time to disengagement were significantly associated with greater weight loss; however, consistency and time to disengagement for self-monitoring of physical activity were not associated with weight change. Finally, although most participants disengaged with at least one of the digital tools for self-monitoring at some point during the 6-month intervention, over half of those who disengaged re-engaged in self-monitoring of weight (30/45, 65%) and physical activity (21/35, 60%) for at least 1 week; however, <40% (18/46) re-engaged in self-monitoring of dietary intake.

The results of this study have several important implications. First, patterns of consistency and disengagement from self-monitoring with digital technologies were different by behavior, with participants demonstrating significantly greater consistency and longer time to disengagement for self-monitoring of physical activity compared with dietary intake and weight. Similar patterns were also seen in a recent study by Butryn et al [35] and may be related to differences in participant burden when using digital self-monitoring tools. Passive self-monitoring tools (such as the wearable device used to measure physical activity in this study) can require substantially less effort compared with more active methods (eg, the use of a website or smartphone app to record dietary intake, which can take up to 15-20 minutes each day [36]). The increased effort and time burden associated with active self-monitoring may thus adversely affect participant consistency and engagement. Indeed, other research has also demonstrated higher rates of engagement for behaviors that are passively versus actively self-monitored [37,38]. In addition, although self-monitoring weight via smart scales does not represent a similar time burden as self-monitoring of dietary intake, engagement may be impacted by other barriers such as negative emotions. Research has shown that daily self-weighing does not cause adverse psychological outcomes [39-41] but that some individuals may avoid the scale because of shame or denial [42]. For example, 1 study found that individuals tended to avoid self-weighing after a day with above their average dietary intake [43]. Thus, self-monitoring of both dietary intake and weight via digital tools may represent higher-burden behaviors compared with self-monitoring of physical activity via digital tools, which may have adversely influenced participant engagement.

Conversely, a certain level of burden may be necessary for behavior change. A study by Turner-McGrievy et al [44] found that participants who used more passive methods for dietary self-monitoring (eg, photo-based apps and wearable bite counters) reported lower rates of habit formation for self-monitoring dietary intake than participants who entered foods or drinks consumed via smartphone apps. Another study by Silberman et al [45] found that participants in a digital weight management program who weighed themselves using traditional, in-home scales and then manually entered those weights into the study website lost more weight over a 12-month period compared with participants who used smart scales to automatically sync weights with the study website. It may be that manual tracking of some self-monitoring outcomes (eg, dietary intake and weight) may be necessary to improve awareness of the target outcome and promote behavior change. Therefore, determining other ways to increase adherence to digital self-monitoring tools, beyond alleviation of burden, may be important for future weight management research.

Accordingly, the results of this study demonstrated that provision of additional interventionist support may also be key for promoting consistency and preventing disengagement with digital self-monitoring tools, especially for higher-burden behaviors such as self-monitoring of dietary intake. These results were consistent with supportive accountability theory, which posits that additional interventionist support can increase adherence to and engagement with mobile health interventions [46]. The intervention phone calls offered to the TECH+PHONE group provided participants with accountability and feedback on progress (coupling encouragement with opportunities to engage in problem solving to overcome barriers experienced in relation to behavior change), which likely supported the higher levels of engagement with dietary self-monitoring tools observed in this study. Although there were no statistically significant differences between TECH and TECH+PHONE participants for consistency and time to disengagement for self-monitoring of physical activity or weight, there was a small or moderate effect size favoring the TECH+PHONE group. It is possible that, as a pilot study, this study was underpowered to detect effects of this size. Thus, future studies with larger samples should investigate whether additional interventionist support can improve engagement with digital tools for self-monitoring weight and physical activity.

On a final note, the results demonstrated suboptimal adherence to self-monitoring across all behavioral targets. Adherence was defined in relation to intervention goals, which included daily self-monitoring of dietary intake, weight, and physical activity (ie, 7 out of 7 days each week). Alternatively, there may be important thresholds below this level that represent clinically meaningful adherence to self-monitoring. Little research, however, has been conducted to establish these thresholds, and evidence from the weight maintenance literature suggests that clinically meaningful thresholds may differ by behavior. Peterson et al [16] found associations between less weight regain and greater consistency of self-monitoring dietary intake when consistency was defined as self-monitoring intake on ≥3 days per week. Brockmann et al [17] found that greater consistency of self-monitoring weight was only significantly associated with less weight regain when defined as ≥6 days of weight self-monitoring. No studies have investigated clinically relevant thresholds for adherence to self-monitoring of physical activity during the maintenance period after initial weight loss programs, and no studies have examined thresholds for adherence to any of these behaviors during initial weight loss programs. Moving forward, it is important for future studies to identify these minimum thresholds to support the development of clinically meaningful definitions of adherence.

### Strengths and Limitations

This study was the first to examine the effect of providing additional phone-based interventionist contact on engagement with digital tools for the self-monitoring of dietary intake, physical activity, and weight, independently. A major strength of this study was the use of objective data from the Fitbit Zip digital pedometers and Fitbit Aria smart scales, which reduced the risk of retrospective recall bias. Moreover, issues related to missing data from nonreturn of paper self-monitoring records were minimized because of the collection of self-monitoring data in real time, directly from these devices and the Fitbit website or smartphone app.

This study also had a number of important limitations. First, disengagement was defined as a 2-week period in which intervention self-monitoring goals were not met; however, a prolonged period of adherence to self-monitoring before disengagement does not ensure that participants were adequately *engaged* with the digital self-monitoring technology or even fully adherent to the full process of self-monitoring (ie, observing change in a target behavior to assess progress toward a goal). For example, a participant could have stepped on the scale for the sake of the study without looking at their weight or have worn the activity monitor without paying attention to their step count. Unfortunately, more precise data on engagement were not collected during this study; future studies should attempt to better disentangle the constructs of adherence versus engagement. Second, as the parent study was conceptualized as a pilot trial, the sample size of both the TECH and TECH+PHONE groups were small and predominately consisted of women who identified as White and non-Hispanic. Thus, the results may not generalize to broader populations, including groups disproportionally burdened by obesity (eg, adults who identify as Black and Hispanic) [47]. Additional research on engagement with digital tools in larger and more diverse samples, especially those including larger numbers of men and individuals from backgrounds historically underrepresented in clinical research, is critical. Third, the parent trial did not include measures assessing participants’ history of or comfort with using digital self-monitoring tools, which could have influenced adherence to self-monitoring in this study. However, even though the data on the exact number of technical errors are not available, few technical issues were experienced and were easily addressed. Finally, this study did not investigate week-to-week associations between changes in weight, dietary intake, and physical activity and later changes in self-monitoring behaviors; however, some emerging research suggests that there may be important bidirectional associations between self-monitoring and changes in weight and weight-related behaviors [48]. Future studies with larger samples should characterize the nature of these associations across time.

### Conclusions

This study examined consistency with and disengagement from digital tools for self-monitoring of weight and weight-related behaviors in a technology-based weight management intervention. The results demonstrated that patterns of consistency and disengagement varied by behavior, such that participants demonstrated greater consistency and longer time to disengagement from self-monitoring of physical activity compared with weight and dietary intake. Furthermore, the results demonstrated that the provision of additional phone-based interventionist contact led to greater consistency in self-monitoring of dietary intake. Importantly, greater consistency and longer time to disengagement from self-monitoring of dietary intake and weight were associated with greater weight loss during the 6-month intervention. Given the key role of self-monitoring for weight loss and weight loss maintenance, future studies should replicate these results in larger, more generalizable samples and focus on identifying novel methods to improve individuals’ engagement with digital self-monitoring tools.

## Abbreviations

REDCap: Research Electronic Data Capture
TECH: technology-based self-monitoring tools
TECH+PHONE: technology-based self-monitoring tools plus additional phone-based intervention
